# Clinical Perspective and Translational Oncology of Liquid Biopsy

**DOI:** 10.3390/diagnostics10070443

**Published:** 2020-06-30

**Authors:** Diego Fernández-Lázaro, Juan Luis García Hernández, Alberto Caballero García, Aurora Caballero del Castillo, María Villaverde Hueso, Juan Jesús Cruz-Hernández

**Affiliations:** 1 Department of Cellular Biology, Histology and Pharmacology, Faculty of Health Sciences, University of Valladolid, Campus de Soria, 42003 Soria, Spain; maria.v.hueso@gmail.com; 2 Cancer Research Centre, University of Salamanca, 37007 Salamanca, Spain; jlgarcia@usal.es; 3 Institute of Biomedical Research of Salamanca, University Hospital of Salamanca (IBSAL), 3007 Salamanca, Spain; jjcruz@usal.es; 4 Department of Anatomy and Radiology, Faculty of Health Sciences, University of Valladolid, Campus of Soria, 42003 Soria, Spain; albcab@ah.uva.es; 5 Oncology Service, University Hospital of Salamanca, 37007 Salamanca, Spain; aurora95_narros@hotmail.com

**Keywords:** liquid biopsy, cancer detection, clinical practice, molecular profile, minimal residual disease, response monitoring, early detection, resistance mechanisms, translational oncology

## Abstract

The term liquid biopsy (LB) refers to the study of circulating tumor cells, circulating tumors nucleic acids free of cells or contained in exosomes, and information about platelets associated with tumors. LB can be performed in different biofluids and allows the limitations of tissue biopsy to be overcome offering possibilities of tumor identification reflecting in real time tumor heterogeneity. In addition, LB allows screening and early detection of cancer, real-time monitoring of therapy, stratification and therapeutic intervention, a therapeutic target and resistance mechanism, and a risk of metastatic relapse. Currently, LB has been shown to be effective for its application in different types of tumors including lung, colorectal, prostate, melanoma, breast and pancreatic cancer, by the determination and identification of biomarkers that with a high probability have the potential to change the way in which medical oncology could predict the course of the disease. These biomarkers make it possible to capture the heterogeneity of the cancer, monitor its clonal evolution, indicate new treatments or retreatments and evaluate the responses to different evolutionary and/or therapeutic pressures in the cancer disease.

## 1. Liquid Biopsy

Cancer disease is an active pathology as a consequence of clonal expansion responsible for the progression of biological processes that trigger tumor development [1]. The ontogeny of cancer is in the accumulation of mutations originating in each of the mechanisms responsible for carcinogenesis. In this way, the evaluation of the genetic mutations related to the tumor could serve as tools in the diagnosis and prognosis. Simultaneous monitoring of pacemakers and treatment linked to these mutations could establish an individualized medicine plan for each patient [2].

Biopsies of tumor tissue are the protocoled way to diagnose cancer. The molecular determination tests and the selection of personalized therapies use the tissue biopsies as a guide. Tissue biopsy consists of the collection of cells from the human body and is a procedure for easy and frequent monitoring of oncogenic mutations [3]. However, it presents limitations: (i) high clinical risk for the patient; (ii) high economic cost; (iii) it is a highly invasive and uncomfortable technique; (iv) there are technical limitations associated with tumor localization; (v) there is difficulty in extracting the cellular subpopulations that make up the tumors; (vi) the dissemination of tumor cells to other organs and tissues inaccessible for tissue biopsy; and (vii) an inability to perform serial testing (e.g., after removal of the primary tumor) [3]. To overcome these limitations an innovative tool is the liquid biopsy (LB) which could be a molecular determination technique to assess the heterogeneity of tumors, their detection and monitoring. LB is a modern biomarker analysis tool that uses the body fluids of patients present in non-solid biological tissue (blood, urine, saliva, urine, cerebrospinal fluid, and pleural effusion). Biomarkers are circulating tumor cells (CTCs), cell-free circulating nucleic acids, exosomes and platelets associated with tumors. All of them are released into the peripheral blood from primary tumor and/or metastatic deposits [3]. These characteristics of the LB establish it as a minimally invasive method, of lower cost than tissue biopsy and without the use of surgery. Some clinical applications for the LB in medical oncology have been suggested, such as determinate molecular profile, diagnosis, response monitoring and early detection of resistance mechanisms, early detection and screening, determination minimal residual disease. Also, this could be used in lung, colorectal, prostate, melanoma, breast and pancreatic cancer [3,4].

**Figure 1:**
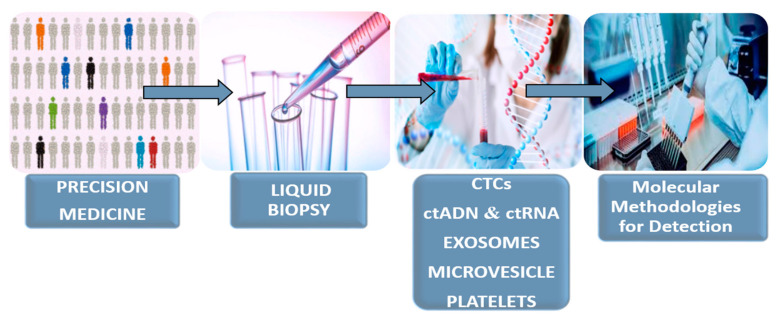
Operative flow of the liquid biopsy: individualized therapeutic strategy for capture dynamics of the tumoral disease.

However, technological, instrumental and scientific difficulties pose a challenge to LB until it is used in the clinic in a standardized way. For this reason, it is mandatory to validate and establish standardized working protocols for all stages that make up LB (Figure 1). In addition, it is necessary to evaluate whether the biomarkers found in the biofluids reproduce the results of tissue biopsies [5,6].

In this sense, LB is the analysis of the circulating biomarkers, which describe more completely the dynamics of the tumoral disease in real time and which contain tumoral materials from different sites of the disease in the body, requiring different molecular detection technologies: quantitative polymerase chain reaction (q-PCR), BEAMing, safe-sequencing system (Safe-SeqS), cancer personalized profiling by deep sequencing (CAPP-Seq), digital polymerase chain reaction (dPCR), copy number aberrations (CNAs) or point mutations by sequencing the entire genome (whole-genome sequencing, WGS) or sequencing the entire exome (whole-exome sequencing, WES) and tagged-amplicon deep sequencing (TAmSeq) [7] (Table 1). These techniques evaluate mutations in the primary tumor and are extremely sensitive, since mutations can be detected at an allele frequency with high specificity showing complete information about the tumor genome and permit to identify new changes that occur during treatment of the tumor and no prior information about the genome of the primary tumor is required [8,9]. These molecular technologies allow positioning the LB as an indispensable clinical test for personalized medicine through the analysis of its biomarkers for prognostic and predictive purposes by non-invasive means, which in the near future will represent a change in the paradigm of molecular biological understanding and the heterogeneity of tumors [10]. Consequently, the LB monitors in real time the dynamics of the cancer disease [11].

## 2. Clinical Perspective of Liquid Biopsy

**Figure 2:**
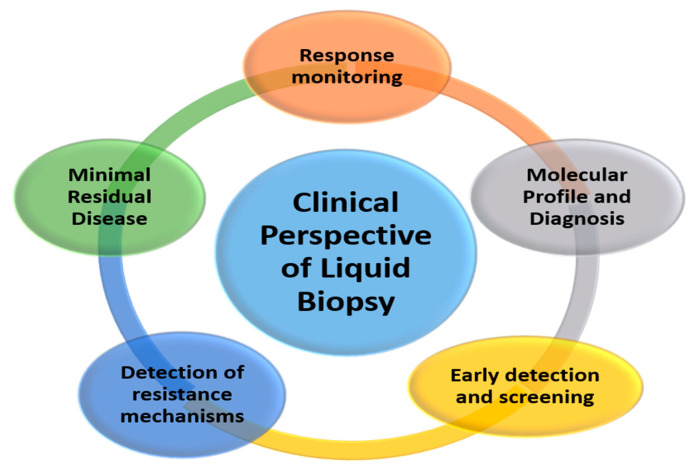
Potential clinical applications of liquid biopsy.

The possibility of performing a molecular perl as simple as a blood extraction, which is non-invasive and can be repeated as many times as necessary, opens the door to a significant number of clinical applications that are developing rapidly and will be used in the coming years [3] (Figure 2).

### 2.1. Molecular Profile and Diagnosis

Cancer treatment is based on histological molecular profile and diagnosis in order to select the most appropriate therapies for each patient’s clinical situation. The molecular study of tumor products by LB is mainly based on ctDNA but also CTCs or exosomes that allow the molecular and evolutionary characteristics of the tumor to be established, which will determine its tumor heterogeneity [12]. In addition, LB is increasingly used to generate information on the transcriptome, epigenome, proteome and metabolome. This makes it possible for CTCs and ctDNA to become a clinical and/or research platform to overcome the limitations of current methods of diagnosis and follow-up of cancer patients [6]. For these reasons, LB is a necessity in precision oncology.

Some clinical trials have used cDNA-based molecular profiling as a tool for treatment selection in solid tumors such as lung cancer, prostate cancer and breast cancer [13]. In lung non-small cell lung cancer (NSCLC), the ctDNA determination of mutations of the epidermal growth factor receptor (EGFR) gene including the T790M-resistant allowed to selection the treatment between two drugs (erlotinib or osimertinib) [14]. The ctDNA assay for the determination of the anaplastic lymphoma kinase (ALK) gene mutation (ALK F1174C) in small cell carcinoma of the prostate, establishes a molecular profile that could focus the treatment on the use with ALK inhibitor (alectinib) [15]. In metastatic breast cancer, the use of ctDNA in determining the presence of estrogen receptor 1 (ESR1) mutations was the molecular profile used in the Phase III clinical trials PALOMA-3 and SOLAR-1. These clinical trials evaluate the use of CDK4/6 inhibitors in conjunction with endocrine therapy with Fluvestrant [16]. Thus, the use of ctDNA sequencing seems be adequate in the identification of patients with somatic mutations associated with increased risks of cancer.

In addition, the use of CTCs to establish the molecular profile and diagnosis based on the enumeration and characterization of the CTC. In breast cancer, CTCs were used to select the first line of treatment and established the poor prognosis of the initial number of CTCs [17]. Similarly, in prostate cancer the positive determination androgen receptor splice variant 7 (AR-V7) in CTCs showed a better response to taxanes on display, superior overall survival, and resistance to enzalutamide and abiraterone [18]. We believe that the determination of the molecular profile could improve the ability to diagnose a patient, however, more prospective clinical trials to demonstrate the clinical utility of a LB cancer screening test instead of standard screening programs are essential conditions.

### 2.2. Response Monitoring and Early Detection of Resistance Mechanisms

LB is an advance in achieving the clinical objectives set because it allows the effectiveness of treatments to be evaluated using new biomarkers in addition to routine clinical indicators (overall survival, or progression-free survival of the disease). These biomarkers serve as a substitute for a clinically significant end-point, which is expected to predict the effect of a therapeutic intervention, by earlier and more manageable criteria in both conventional clinical and clinical trials [6].

In early-stage cancers, the main problem is identifying patients at risk of relapse. These patients may benefit from early surgical or radiotherapy treatments. Patients with pT1-T2 and pN0-N1 stage breast cancer from the National Cancer Database and in the multi-center Phase III clinical trial SUCCESS who were identified as having CTCs were associated with overall survival and disease-free survival by benefiting adjuvant surgery or radiotherapy after breast-conserving surgery [19].

The monitoring of pharmacological therapy using LB makes it possible to determine the tumor phenotype over time, i.e., the evolution of the tumor that is essential when a treatment is administered. Therefore, it allows its effectiveness to be monitored, as well as the possible resistances that could appear in the course of the treatment. The detection of these resistant cell phenotypes allows not only unnecessary toxicities to be avoided, but also the treatment to be individually adjusted before the disease progresses [5]. In addition, the different drugs used against cancer have a very low therapeutic index (therapeutic dose/toxic dose ratio), so the precision must be very high and, additionally, it must have continuity over time. This poses a challenge due to the molecular, cellular, tissue and clinical complexity of cancer disease, which could be resolved by the so-called LB, which conceptually meets all these requirements. This could radically change the diagnostic and therapeutic approach to cancer, as well as the clinical monitoring of this disease [4]. 

Also, ctDNA analysis has a usefulness in detecting resistance mechanisms [3]. The determination of genomic aberrations in ESR1, mitogen-activated protein kinase (MAPK), Retinoblastoma gene 1 (RB1), Thr790Met (T790M), Ki-ras2 Kirsten rat sarcoma viral oncogene homolog (KRAS) and proto-oncogene B-Raf (BRAF) genes that appear after chemotherapy treatments in breast cancer with aromatase inhibitors [20] or CDK4/6 inhibitors [21], lung cancer with osimertinib [22], colon cancer with cetuximab [23] and melanoma with BRAF inhibitors [24] may provide a better strategy to delay drug resistance. In general, the LB allows the monitoring of the response and is able to establish early resistance mechanisms which could be achieved up to 10 months prior to radiological progression. This implies the possibility of indicating new treatments or retreatments, allowing the monitoring of clonal evolution and responses to different evolutionary and/or therapeutic pressures, for example, colorectal or breast cancer [10].

### 2.3. Minimal Residual Disease

The minimal residual disease (MRD), is a clinical state in which the patient has tumor cells disseminated from the primary lesion to distant organs without clinical or radiological signs of metastasis or residual tumor cells [5]. The detection of minimal residual disease (MRD) represents an unsolvable problem in conventional medical oncology. One of the advantages of LB is that it potentially allows MRD. This is especially important in two clinical situations: in adjuvant treatments or after intended surgery or in early detection of relapses [5]. Therefore, the detection of cell-free circulating DNA (cfDNA) could be useful, as demonstrated by Tie et al. [25] in patients with stage II/III colon and rectal cancer where there is no standardized method and/or definition or consensus on the population at risk of micrometastasic disease. In this study it was observed that the percentage of recurrence was 10 times higher when ctDNA was detected after surgery [10]. 

The possibility offered by the detection and characterization of MRD using CTCs was reflected in the fact that the presence of CTCs was a predictive marker of shorter disease-free survival (DFS) and overall survival (OS) in breast cancer patients after Phase III clinical trial SUCCESS study [26]. Also, CTCs detection after surgery might be a predictive marker of benefit from adjuvant radiotherapy because in patients with CTCs detected before adjuvant therapy radiotherapy was associated with longer OS, loco regional relapse-free survival and disease-free survival (DFS) [19]. Also, colorectal cancer patients, who were found to have CTCs years after surgery had a worse prognosis and increased recurrence [27]. The lower number of post-treatment CTCs in NSCLC patients was correlated with a higher shorter disease-free survival (DFS) (*p* < 0.001) and overall survival (OS) (*p* = 0.009) [28]. Thus, CTCs counts could early surrogate end point that predicts survival in patients with cancer [5].

### 2.4. Early Detection and Screening

The last promise, which may come true in a short space of time, about the usefulness of LB is the detection of early disease, long before it appears clinically or radiologically. Until then, work must be done to improve sensitivity in the detection of ctDNA in asymptomatic patients with very early stage tumors [29].

In early stage cancers, LB allows cancer patients to be discriminated from healthy controls, thus screening besides detecting early cancers, and it also permits locating the organ of origin of the tumor. In this sense, the CancerSEEK blood test detect eight biomarkers of circulating proteins and tumour-specific mutations in the ctDNA. In a study of 1000 patients previously diagnosed with cancer and 850 healthy control individuals, CancerSEEK detected cancer with a sensitivity of 69 to 98% (depending on the type of cancer) and 99% specificity [30]. Also the detection cancer-derived (Epstein Barr–virus) EBV DNA in plasma has proven to be a useful screen for early detection of nasopharyngeal carcinoma in asymptomatic subjects, with high sensitivity and specificity analysis of plasma Epstein–Barr virus DNA to screen for nasopharyngeal cancer [31]. 

## 3. Translational Oncology Application of Liquid Biopsies in Cancer Therapy

The effectiveness of this tool has been demonstrated in different tumors including lung, colorectal, prostate, melanoma, breast and pancreatic cancer, among others [32]. 

### 3.1. Lung Cancer

Lung cancer, especially NSCLC, is the world’s leading cause of cancer death. Lung cancer has evolved from virtually a single disease and a single treatment for all to be the paradigm of modern medical oncology, with different molecular diagnoses that condition targeted treatments. These personalized strategies need recent tissue that in this disease is usually insufficient in many cases, however increased knowledge of the molecular biology of cancer, together with the development of techniques with highly sensitive detection technologies for molecular analysis based on PCR or next-generation sequencing (NGS) in plasma could be the ideal complement to conventional targeted therapies [32]. The immunotherapy based on antibodies against the Programmed Death-ligand 1 (PD-L1) and EGFR tyrosine kinase inhibitors are some targeted therapies in lung cancer. Thus, the difficult access to the tumor (because of its location) and the risk for the patient of tissue biopsy techniques, limit the compression of lung cancer. LB, minimally invasive technique, would solve this problem and could detected the expression of PD-L1 in CTCs or in white blood cells, although with the limitation of the isolation of these CTCs and the concordance with tissue, and the clinical impact of the same. In lung cancer, the tumor mutational load is suggested as being a predictive biomarker for immunotherapy, it being possible to carry this out in plasma and identifying patients who would benefit in the second line of atezolizumab [33]. Also, in 37 advanced NSCLC patients, used the analysis of CTCs and ctDNA, was positive for all samples for the detection of EGFR T790M mutations, this multi-marker analyses may achieve a better clinical result [7]. 

**Figure 3:**
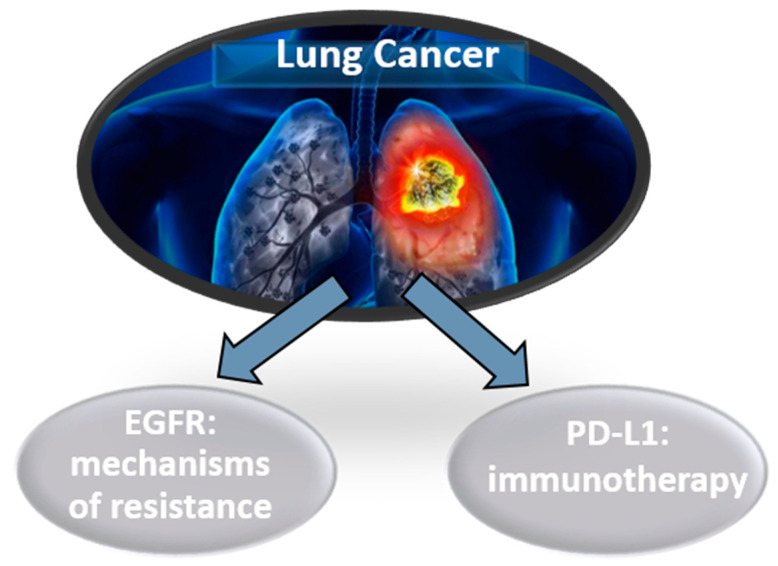
Liquid biopsy’s biomarkers for optimal treatment in lung cancer.

LB is, at the present time, a complementary tool to tumor biopsy in molecular diagnosis and guiding the targeted treatment of advanced lung cancer. The study of genetic alterations/mutations in LB requires more sensitive techniques than those normally used for the characterization of tumor biopsies [3]. Thus, the most frequent application of LB in lung cancer is the determination of mutations in EGFR to identify genomic mechanisms of resistance to targeted therapy and detected PD-L1 expression for immunotherapy (Figure 3). In addition, the detection of ctDNA after radical treatment in early stages of lung cancer could predict the probability of relapse in follow-up. However, studies in liquid biopsy using CTCs, exosomes or educated platelets are promising, but have not yet been incorporated into routine care.

### 3.2. Colorectal Cancer

Colorectal cancer is characterized by having a very heterogeneous and variable genetic profile, which results from the constant acquisition of new mutations throughout tumor development. In this way, the detection of tumor mutations through LB may be of great help in the monitoring of the disease. In this connection RAS is one of the most frequently mutated oncogenes in human cancer but the frequency and distribution of RAS gene mutations are not uniform. For instance, KRAS is the isoform most frequently mutated, which constitutes 86% of RAS mutations. KRAS-4B is the dominant isoform in human cancers, and it is present in approximately 90% of pancreatic cancers, 30% to 40% of colon cancers, and 15% to 20% of lung cancers, mostly NSCLC. It is also present in biliary tract malignancies, endometrial cancer, cervical cancer, bladder cancer, liver cancer, myeloid leukemia and breast cancer [3,34]. However, despite its well-recognized importance in cancer malignancy, there are no approved therapies for KRAS mutant cancer. Recent LB studies have aroused renewed interest in the development of KRAS inhibitors either directly towards mutant KRAS or against the crucial steps required for KRAS activation [6,10]. 

In this way, the methodology used in liquid biology as NGS and dPCR technologies offer different advantages and disadvantages that make each approach suitable for specific clinical applications of ctDNA for RAS (Table 2). NGS panels offer the advantage of simultaneously detecting multiple molecular alterations, but at a higher cost and lower sensitivity. NGS could be a key tool for simultaneous screening of multiple molecular alterations for therapeutic decisions in tumors with several therapies or monitoring the occurrence of multiple coexisting resistance mechanisms (during anti-EGFR therapy in colon cancer), as well as for the discovery of new targets. By contrast, dPCR offers increased sensitivity in detecting mutant alleles, reaching a detection threshold of 0.001 ng/mL of mutKRAS alleles, making it an appropriate technique for the detection of MRD or the detection of specific mutations in RAS [3,12,25].

Tumor genetic analysis is not only useful for monitoring the disease but also for determining the response to treatment, as is the case with anti-EGFR therapy in which only patients with the non-mutated KRAS gene respond [34]. The genotyping studies establish a concordance of more than 90% between KRAS tests in tumor tissue and ctDNA, making it possible to test in ctDNA LB as valid for tissue tests in patients with colorectal cancer [35]. Thus, the detection of tumor mutations through the BL, contribute to the monitoring of the disease and determine the response to treatment as is the case of anti-EGFR therapy in which only patients with the KRAS gene without mutations respond [34]. Resistance has also been determined by KRAS mutations in the blood of patients treated with anti-EGFR antibodies up to 10 months before radiological progression. Even if highly sensitive sequencing techniques are used, KRAS mutant subclones are detected in plasma before starting treatment [20]. 

Moreover, possible retreatment with anti-EGFR drugs is currently being evaluated in the clinic. The ctDNA could be used to measure plasma RAS mutations and guide the administration of drug breaks and retreatment with anti-EGFR therapy [36]. Thus, the detection of the molecular mechanisms of resistance in ctDNA can guide clinicians to avoid continuing with ineffective treatments and decide on personalized treatment after progression to anti EGFR drugs. Clinical trials of ctDNA include microsatellite instability (MSI) testing and evaluation of tumor tissue mutational burden (TMB) in ctDNA to identify patients with metastatic high microsatellite instability (MSI-H) colorectal carcinoma who can guide immunotherapy and assess response in serial extractions of ctDNA [37]. Finally, the usefulness of ctDNA for detecting MRD in patients with colorectal cancer has also been described [38] and quantification of ctDNA correlates with tumor load and allows the use of evaluate early response to cancer treatment [34]. 

In summary, the main clinical applications of LB in colorectal cancer include: molecular diagnosis for treatment decisions, determination of tumor burden as a surrogate marker for early response to treatment, monitoring of resistance mutations to targeted treatment, and detection of minimal residual disease after cancer surgery. 

### 3.3. Pancreatic Cancer

In pancreatic cancer, LB is presented as a very advantageous technology given the anatomical complexity of the tissue, as well as the difficulty in accessing it. This situation means that a small number of patients with pancreatic cancer survive more than 5 years, partly because the majority of patients identify their tumor status when the disease is at an advanced stage [39]. In the detection of pancreatic ductal adenocarcinoma, blood tests to detect mutations in ctDNA, specifically in the KRAS gene, have been combined with protein biomarkers that include carcinoembryonic antigen (CEA), carbohydrate antigen 19-9 (CA19-9) and cancer antigen 125 (CA125) [40]. 

Cohen et al. [40] have showed that the analysis of routine protein biomarkers (carcinoembryonic antigen (CEA); carbohydrate antigen 19-9 (CA19-9); cancer antigen 125 (CA125)) together with the determination of KRAS gene mutations (using ctDNA) had greater diagnostic power than any single marker. In addition, only one control sample was positive for either KRAS or protein biomarkers (99.5% specificity). In this study [40], using a combination of biomarkers, distant metastasis was detected in two thirds of the patients at the time of surgery. Moreover, plasma KRAS mutations (30% of patients) were confirmed in tissue obtained from the primary tumor. These results may be a promise and could establish that the joint use of ctDNA and proteins together could be useful for the early detection of many types of cancer.

### 3.4. Breast Cancer

**Figure 4:**
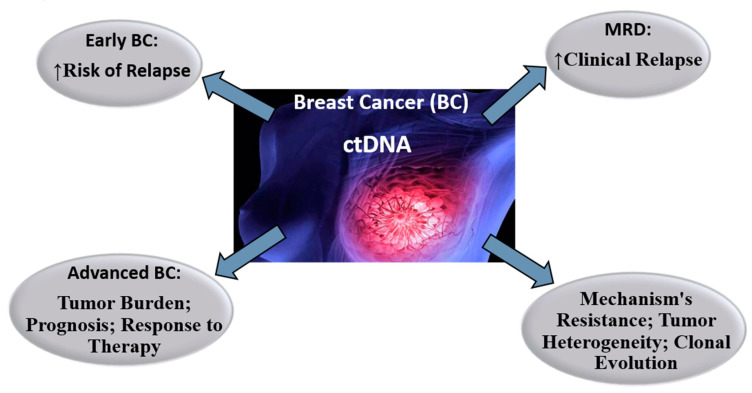
Perspectives on the role of ctDNA in the therapeutic approach to breast cancer.

After breast cancer patients have been diagnosed, two fundamental clinical objectives must be met, such as identifying who should or should not receive adjuvant treatment, especially chemotherapy, and which is the most appropriate therapy or combination of therapies for a given patient. The traditional way of doing this is with a combination of clinical methods and routine pathological techniques. LB, with plasma detection of ctDNA, could meet these clinical objectives (6) (Figure 4). 

Breast cancer has pioneered the use of the biomarker HER2, which is over-expressed in 10% to 25% of breast tumors causing tumor growth, through the stimulation of MAPK and PI3K/AKT signaling pathways, resulting in metastasis and, therefore, a poor prognosis for the patient. Also the deformation of cell membranes, by overexpression of HER2 could develop aberrant cell proliferation [41,42]. HER2 measurement was originally proposed as a prognostic biomarker and is now mandatory in all new cases of invasive breast cancer and in recurrent or metastatic lesions. In addition, HER2-targeted treatments have transformed the outcome of HER2-amplified cancers. In this sense, amplifications can be “*acquired*” and “*lost*” through tumor progression and treatment, representing a substantial challenge for the concept of personalized therapy against breast cancer, because it is necessary to know the state of heterogeneity of the disease at each moment. Therefore, the detection of the HER2 gene biomarker responds to all patient needs. In medical oncology, it assumes that detecting HER2 amplifications becomes part of routine practice, which will require continuous DNA extraction, to determine the genetic profile at that time of the breast cancer. Tissue biopsy usually takes samples from a single area of the tumor, requires repeated biopsies with all associated risks, and can be technically difficult depending on the sites of the cancer relapse [42,43]. Ideally, to overcome these limitations and to allow repeat sampling, the presence of HER2 amplification can be diagnosed non-invasively by LB. This highlights the potential usefulness of an accurate and sensitive system based on ctDNA detection, such as dPCR assays, for accurate quantification of the number of copies of the HER2 gene in the DNA extracted from the blood. Recently, several studies [29,42,43] have demonstrated the clinical utility of dPCR in determining HER2 mutations in breast cancer. Although overexpression of HER2 appears to be necessary for optimal response to anti-HER2 therapy, many patients do not respond to therapy with trastuzumab monoclonal antibody and those who do (30%) end up developing resistance after a long period of time. This implies that new biomarkers, different to HER2, will monitor responses to trastuzumab or other anti-HER2 therapies [44]. 

On the other hand, PIK3CA activated by its own receptor or by insulin growth factor 1 (IGF-1) stimulates the proliferation of neoplastic cells in breast cancer. The concordance of PIK3CA mutations is demonstrated in tumor and plasma samples in breast cancer that have been found to be elevated in both [45]. The use of LB in the detection of PIK3CA mutations has demonstrated a sensitivity of 85–90% and a specificity of 98% in metastatic breast cancer patients with positive tumors to the PIK3CA mutation [45]. Takeshita et al. [46] have demonstrated that the H1047R, E545K and E542K PIK3CA ctDNA mutations in early-stage triple-negative breast cancer had a significant prognostic value (negative) on survival. In addition, patients with a higher level of PIK3CA mutant detected by plasma ctDNA in breast cancer showed a worse prognosis. Moreover, in the BELLE-2 assay [47] (buparlisib plus fulvestrant), detection of PIK3CA mutations in plasma leads to a statistically significant improvement in progression-free survival (7.0 vs. 3.2 months). These results [45,46,47] together suggest that the identification of genomic PIK3CA alterations in plasma will provide a prediction of clinical response. The use of dPCR in breast cancer for the determination of PIK3CA of plasma ctDNA that is concordant with the status of both tumor genes, allowing to know the intertumoral and intratumoral heterogeneity at each time of the evolution of breast cancer which is an advantage in the use of this technique of dPCR [48,49]. 

### 3.5. Prostate Cancer

Most studies of CTCs in urologic neoplasms have been performed in prostate cancer initially only quantified as an objective, but their characterization and studies of their mechanical properties are now necessary. Even other elements that form the LB have been incorporated into studies in prostate cancer as ctDNA or tumor-derived proteins [50]. In the study by Danila et al. [51], they counted CTCs in 120 patients with castration-resistant metastatic prostate cancer using CellSearch^®^ and found that the number of CTCs, treated as a continuous variable, was inversely proportional to overall survival, with the combination of CTCs count, prostate-specific antigen (PSA) and albumin being a better predictor of survival than any of these variables individually. Patients with bone metastases had more CTCs than patients with soft-tissue metastases, and patients who had previously received chemotherapy also had more CTCs.

In one of the first and most important works in prostate cancer, de De Bono et al. [52], were able to find CTCs in 231 of 276 patients with castration-resistant metastatic prostate cancer. In this study they observed that patients with 5 CTCs had significantly worse survival than those with <5 CTCs (11.5 months vs. 21.7 months, HR 3.3, *p* < 0.0001), with the cut-off point of 5 CTCs being a better predictor for overall survival at one year than serum PSA. On the other hand, patients who had <5 CTCs and increased to 5 CTCs also had a worse prognosis, while patients who had 5 CTCs and decreased to <5 CTCs improved it. This was confirmed in another phase 3 study, SWOG0421 by Goldkorn et al. [53]. In this study the patients received Docetaxel with or without delay, and separating again through the cut-off point of 5 CTCs differences were observed; with <5 CTCs they had 26 months of median overall survival, while with 5 CTCs 13 months were observed (HR 2.74, IC 95% 1.72–4.37). And the increase to 5 CTCs in the first 3 weeks after starting treatment showed worse survival (HR 2.55, 95% CI 1.04–6.24). Although there are other studies where the cut-off point was 4 CTCs, the cut-off point of 5 CTCs was mostly used.

Scher et al. [54] described how the dynamic enumeration of the number of CTCs at certain times of disease (CTCs 5 or <5 at weeks 4, 8, and 12 after initiation of treatment) were associated with the risk of death from prostate cancer and that the number of CTCs taken as a continuous variable also correlated with the evolution of these patients. In the COU-AA-301 study [55] as a secondary objective, the number of CTCs was evaluated as a surrogate for overall survival. This study was a double-blind, randomized phase 3 clinical trial comparing abiraterone acetate plus prednisone versus prednisone alone in patients with castration-resistant metastatic prostate cancer (CPRCm) progression to docetaxel. They found that the number of CTCs combined with the serum lactate dehydrogenase (LDH) value met Prentice’s criteria to be a surrogate for overall survival.

On the other hand, ctDNA may also be a useful biomarker, in the Wyatt et al. [56] have detected ctDNA in serial samples (3 months before and after treatment) of 65 patients with CPRCm treated with Enzalutamide the aberrations in the androgen receptor (AR) gene detected in the ctDNA prove to be predictors of response to androgen-deprivation therapy. The LB can provide information to determine the existence of mutations at the AR level. Evidence suggests that AR variants contribute to the progression of prostate cancer by inducing the transition from epithelial to mesenchymal stem cells, allowing for stem cell migration and expansion [57]. In prostate cancer, the determination of the biomarker of the androgen receptor splice variant 7 (AR-V7) has been related to a high profile of aggressiveness, greater resistance to castration and a shorter time of relapse after radical prostatectomy. In new drugs in castration-resistant prostate cancer patients such as Enzalutamide and Abiraterone the presence of AR-V7 is associated with increased drug resistance. Thus, AR-V7 could serve as a prognostic, predictive and selection biomarker for some treatments in advanced prostate cancer [58]. The expression of AR-V7 has been determined by proteins present in the plasma of prostate cancer patients by means of a novel automated capillary nano-assay (ProteinSimple) [18].

### 3.6. Melanoma

Melanoma accounts for only 10% of all skin tumors and is the skin tumor with the highest morbidity and mortality. Despite therapeutic advances, relapses and survival rates below 15% at 5 years make it essential to incorporate new biomarkers into medical oncology for personalized treatment selection and response monitoring. In this sense, ctDNA as a prognostic factor (in localized and advanced disease) and predictive of response, and could play a determining role in tumor assessment [59]. In localized melanoma, Lee et al. [60] have published a study in patients with high-risk stage II/III melanoma where they correlate the presence of ctDNA after surgery and the risk of recurrence. This is a retrospective analysis that included 161 patients with BRAF V600E or neuroblastoma RAS viral oncogene homolog (NRAS) pQ61L/K mutated melanoma in primary tumor and in which the presence of the same mutations in ctDNA was analyzed within 12 weeks after surgery using dPCR. It is important to note that detection of ctDNA was independent of tumor thickness, ulceration, or the presence of affected lymph nodes and that, therefore, its presence was not dependent on the pre-surgical tumor stage, but can be attributed to the presence of post-surgical micrometastatic disease.

In advanced non-resecalbe melanoma, ctDNA could be a biomarker complementary to lactate dehydrogenase (LDH) and radiologic detection of tumor volume. In this sense, Gonzalez-Cao et al. [61] in patients with metastatic melanoma BRAFV600E mutated in treatment with BRAF inhibitors analyzed ctDNA by digital PCR (sensitivity of 0.005%) and observed that the presence of ctDNA correlated with the clinical stage, being more likely to detect ctDNA in tumors with visceral disease (61–75%) compared with those patients who only presented skin metastases (20%). In addition, ctDNA detection was related to the presence of visceral disease, higher tumor volume measured by positron emission omography - computed tomography (PET/CT) and lower survival regardless of the treatment received (median progression-free survival of 39.1 weeks vs. 102.2 *p* = 0.002). Mc Evoy et al. [62] have reported a study that correlates the presence of ctDNA in patients with metastatic melanoma with tumor volume and prognosis. This study showed that tumors presenting a mutation of BRAF V600 in tissue were determined to be the same mutation in ctDNA by digital PCR. Another recent study conducted by Valpione et al. [63] in patients with metastatic melanoma in the first line of systemic treatment. A direct correlation was described by cfDNA and tumor volume (0.52, *p* < 0.001, as well as between changes in cfDNA concentration and tumor volume on the first response assessment CT (0.49, *p* = 0.002). It is important to note that this correlation was maintained regardless of the site of metastasis and treatment received.

Finally, Bidard et al. [64] have studied ctDNA and CTCs as a prognostic value in patients with metastatic uveal melanoma. The analysis was performed by PCR showing that the sensitivity for detection of ctDNA was superior to the detection of ≥1 CTC (84% of patients vs. 30%). Both were associated with high tumor volume and disseminated liver disease, but only the presence of ctDNA was statistically associated with worse disease prognosis. Therefore, we believe that given the technical complexity and low sensitivity of detection of CTCs coupled with the lower ability to detect prognosis, ctDNA could be the tool to be further developed for melanoma patients. With regard to the usefulness of LB as an instrument for monitoring treatment response, ctDNA may be a surrogate marker of tumor volume during systemic treatment in patients with advanced disease. The high turnover of ctDNA with a half-life of only a few hours makes it a real-time information tool for the evaluation of each individual patient [63]. Thus, we can highlight that the quantitative determination of BRAF V600 plasma mutations obtained in ctDNA, from metastatic melanoma patients, could serve to monitor treatment, predictor of response and early determination of resistance to treatment with BRAF/ mitogen-activated protein kinase (MEK) inhibitors [64]. 

## 4. Pitfalls of Using Liquid Biopsy for Precision Medicine

LB is a novel tool that represents a challenge in precision medicine and faces some problems (Table 3), however is possible that its potential clinical applications will be achieved in the immediate future. At the present, the application of LB focusing on the technologies and their analytical/clinical validity. Although many studies have been performed to prove the clinical utility, most were retrospective, so more rigorous studies are required to validate the substantial potential of LB [13]. At the present, there are no studies of clinical use protocoled for the majority of LB’s biomarker in screening, early-stage and advanced cancer. For these reason it will be necessary to validate and standardize the entire process, from obtaining the sample, through its analysis to demonstrating its clinical usefulness [3]. Also, there is the technical complexity of LB, as it is so complex that it is not found in day hospitals with a large flow of patients, and this fact prevents large-scale clinical trials. However, in future, clinical trials are expected to improve patient outcomes and change precision medicine [3]. Also, the molecular biology technology required for the development of LB is currently quite expensive, although possibly as it is used more frequently, costs will decrease so that it becomes more affordable and can be used in daily practice [10].

Along these lines, we believe that it is necessary that clinical trials with inclusion criteria based on LB´s biomarkers detection focus on the maximization of the yield, in order to reach decisions for individualized therapeutic strategy at each stage of the disease and development of targeted drugs designed to eliminate dormant CTCs, maintain them in a quiescent status and use ctDNA as targeted for optimal personalized treatment. LB may be a tool to better monitor the heterogeneity of the cancer and its evolution. However, important clinical validation studies are also required to evaluate and demonstrate the efficacy of the various markers found in liquid biopsy, with (exosomes, ctDNA or CTCs) in clinical settings, but must also demonstrate benefits and improvements in the patient’s disease [3]. Finally, studying the complementarity of the various components of LB, potentially originating from different populations of cancer cells, will open a door to fulfilling all the clinical perspectives of LB, but for this to happen further research studies must be conducted. 

## 5. Conclusions

LB has been shown to be effective for its application in different types of tumors including lung, colorectal, prostate, melanoma, breast and pancreatic cancer, through the determination and identification of biomarkers, extracted with non-invasive techniques, in different biofluids. These biomarkers make it possible to capture the heterogeneity of cancer, monitor its clonal evolution, indicate new treatments or re-treatments and evaluate the responses to different evolutionary and/or therapeutic pressures in cancer disease. Taking the properties of LB into consideration, the use of LB in medical oncology for different types of cancer could actually become a reality.

## Figures and Tables

**Table 1:** Properties of the different molecular detection techniques.

Molecular Detection Techniques	Properties
**quantitative polymerase chain reaction** **(qPCR)**	qPCR, is a laboratory technique of molecular biology based on the polymerase chain reaction (PCR). qPCR is regarded as the ‘gold standard’ in the quantitative analysis of nucleic acids, be it DNA, RNA or micro-RNA molecules. qPCR has high sensitivity, robustness, good reproducibility, broad dynamic quantification range, and very importantly, affordability.
**Safe-Sequencing System** **(Safe-SeqS)**	Safe-SeqS is a Unique Molecular Identifier (UMI) approach to detect rare variants. Safe-SeqS assigns a UMI to each template molecule and amplifies each uniquely tagged template molecule to create UMI families. The abundance of each UMI can be used to distinguish between rare mutations and technical errors, as well as to correct for PCR amplification bias
**CAncer Personalized Profiling by deep Sequencing** **(CAPP-Seq)**	CAPP-Seq is a next-generation sequencing based method used to quantify circulating DNA in cancer. CAPP-Seq is an economical and ultrasensitive method and could be routinely applied clinically to detect and monitor diverse malignancies, thus facilitating personalized cancer therapy.
**Digital PCR** **(dPCR)**	dPCR is a modification of the qPCR method that can be employed to quantify precisely defined nucleic acid targets. The technique is based on the concept of limiting dilutions, which involves the partitioning of a PCR reaction into multiple sub-reactions such that each sub-reaction either contains none or one or more DNA targets. Following thermal cycling, reactions are classified as either positive (target detected) or negative (no target detected), hence providing the basis for a digital output format. By determining the proportion of empty partitions, Poissonian statistics can be applied and the initial number of target molecules present can be estimated.
**Copy number alterations** **(CNAs)**	CNA are somatic changes in chromosome structure that result in gains or losses in copies of DNA sections in somatic tissue, and are prevalent in many cancers. CNA has facilitated the discovery of tumor suppressor genes and oncogenes. Microarray-based CNA assays designed to detect these chromosomes copy number alterations on a high-resolution, genome-wide scale have emerged as a key technology in the genomic era.
**Whole-genome sequencing** **(WGS)**	WGS is a comprehensive method for analyzing entire genomes. Whole genome sequencing WGS has revolutionized the biosciences and proven to be essential and invaluable to the identification of gene functions and their involvement in disease. The feasibility of WGS analysis is under the support of next generation sequencing (NGS) technologies, which require substantial computational and biomedical resources to acquire and analyze large and complex sequence data.
**Whole-exome sequencing** **(WES)**	WES is a genomic technique for sequencing all of the protein-coding regions of genes in a genome (known as the exome). WES provides coverage of more than 95% of the exons, which harbor the majority of the large genetic variants and single nucleotide polymorphisms (SNPs) associated with human disease phenotypes. WES strategy starts by narrowing down the details of variants to be studied by filtering against databases such as HapMap, from the approximately 3.5 million SNPs identified in the human genome project. This focus enables a simpler way for discovery and validation of causative genes and common and rare variants.
**Tagged-Amplicon deep sequencing** **(TAm-Seq)**	TAM-Seq allows targeted sequencing of entire genes to detect mutations in ctDNA. TAM-Seq is based on a multiplex pre-amplification of tiling short amplicons with target-specific primers and initial eminent of the target regions is performed followed by a selective amplification in individual (singleplex) PCRs in order to exclude non-specific products

**Table 2:** Test properties to determine RAS in ctDNA in cancer.

	Next-Generation Sequencing (NGS)	Digital PCR (dPCR)
% Sensitivity	0.1	0.02
Plasma volume (mL)	4	2
Results (week)	2	1
Coverage	High Mutations; Copy-Number Variation; Fusions	Restricted Point Mutations
Advantages	Multiple genes Quantitative	High Sensitivity Quantitative
Disadvantages	High Cost	Restricted Gene Coverage

**Table 3:** Pitfalls of liquid biopsy.

Pitfalls of Liquid Biopsy
Lack of standard and convenient techniques
Test variability and assay sensitivity and specificity
Lack of consensus in technical approaches of choice
Absence of information concerning histology or proliferation index
Absence of information concerning tumor microenvironment
Difficulty in systematically performing immunocytochemistry and in situ hybridation circulating tumor cell (CTCs)
Technological problems in identifying mutations in genes of interest in circulating tumor DNA (ctDNA)
Potentially miss biomarkers expressed in the tumor
Many studies retrospective have been performed to prove the clinical utility

## References

[1] Venesio T., Siravegna G., Bardelli A., Sapino A. (2018). Liquid biopsies for monitoring temporal genomic heterogeneity in breast and colon cancers. Pathobiology.

[2] Hanahan D., Weinberg R.A. (2011). Hallmarks of cancer: The next generation. Cell.

[3] Fernández-Lázaro D., García J.L., Caballero García A., Córdova A., Mielgo-Ayuso J., Cruz J.J. (2020). Liquid Biopsy as Novel Tool in Precision Medicine: Origins, Properties, Identification and Clinical Perspective of Cancer’s Biomarkers. Diagnostics.

[4] Siravegna G., Marsoni S., Siena S., Bardelli A. (2017). Integrating liquid biopsies into the management of cancer. Nat. Rev. Clin. Oncol..

[5] Buyse M., Sargent D.J., Grothey A., Matheson A., De Gramont A. (2010). Biomarkers and surrogate end points—The challenge of statistical validation. Nat. Rev. Clin. Oncol..

[6] Heitzer E., Haque I.S., Roberts C.E., Speicher M.R. (2018). Current and future perspectives of liquid biopsies in genomics-driven oncology. Nat. Rev. Genet..

[7] Lim M., Kim C.-J., Sunkara V., Kim M.-H., Cho Y.-K. (2018). Liquid biopsy in lung cancer: Clinical applications of circulating biomarkers (CTCs and ctDNA). Micromachines.

[8] Beaver J.A., Jelovac D., Balukrishna S., Cochran R.L., Croessmann S., Zabransky D.J., Wong H.Y., Valda P., Cidado J., Blair B.G. (2014). Detection of cancer DNA in plasma of patients with early-stage breast cancer. Clin. Cancer Res..

[9] Czeiger D., Shaked G., Eini H., Vered I., Belochitski O., Avriel A., Douvdevani A. (2011). Measurement of circulating cell-free DNA levels by a new simple fluorescent test in patients with primary colorectal cancer. Am. J. Clin. Pathol..

[10] Bardelli A., Pantel K. (2017). Liquid biopsies, what we do not know (yet). Cancer Cell.

[11] Imamura T., Komatsu S., Ichikawa D., Kawaguchi T., Miyamae M., Okajima W., Ohashi T., Arita T., Konishi H., Shiozaki A. (2016). Liquid biopsy in patients with pancreatic cancer: Circulating tumor cells and cell-free nucleic acids. World J. Gastroenterol..

[12] Maltoni R., Casadio V., Ravaioli S., Foca F., Tumedei M.M., Salvi S., Martignano F., Calistri D., Rocca A., Schirone A. (2017). Cell-free DNA detected by “liquid biopsy” as a potential prognostic biomarker in early breast cancer. Oncotarget.

[13] Rossi G., Ignatiadis M. (2019). Promises and pitfalls of using liquid biopsy for precision medicine. Cancer Res..

[14] Rolfo C., Mack P.C., Scagliotti G.V., Baas P., Barlesi F., Bivona T.G., Herbst R.S., Mok T.S., Peled N., Pirker R. (2018). Liquid biopsy for advanced non-small cell lung cancer (NSCLC): A statement paper from the IASLC. J. Thorac. Oncol..

[15] Carneiro B.A., Pamarthy S., Shah A.N., Sagar V., Unno K., Han H., Yang X.J., Costa R.B., Nagy R.J., Lanman R.B. (2018). Anaplastic lymphoma kinase mutation (ALK F1174C) in small cell carcinoma of the prostate and molecular response to alectinib. Clin. Cancer Res..

[16] El Sayed R., El Jamal L., El Iskandarani S., Kort J., Salam M.A., Assi H. (2019). Endocrine and Targeted Therapy for Hormone-Receptor-Positive, HER2-Negative Advanced Breast Cancer: Insights to Sequencing Treatment and Overcoming Resistance Based on Clinical Trials. Front. Oncol..

[17] Schochter F., Friedl T.W., deGregorio A., Krause S., Huober J., Rack B., Janni W. (2019). Are Circulating Tumor Cells (CTCs) Ready for Clinical Use in Breast Cancer? An Overview of Completed and Ongoing Trials Using CTCs for Clinical Treatment Decisions. Cells.

[18] García J.L., Lozano R., Misiewicz-Krzeminska I., Fernández-Mateos J., Krzeminski P., Alfonso S., Marcos R.A., García R., Gómez-Veiga F., Virseda Á. (2017). A novel capillary nano-immunoassay for assessing androgen receptor splice variant 7 in plasma. Correlation with CD133 antigen expression in circulating tumor cells. A pilot study in prostate cancer patients. Clin. Transl. Oncol..

[19] Goodman C.R., Seagle B.-L.L., Friedl T.W., Rack B., Lato K., Fink V., Cristofanilli M., Donnelly E.D., Janni W., Shahabi S. (2018). Association of circulating tumor cell status with benefit of radiotherapy and survival in early-stage breast cancer. JAMA Oncol..

[20] Fribbens C., Garcia Murillas I., Beaney M., Hrebien S., O’Leary B., Kilburn L., Howarth K., Epstein M., Green E., Rosenfeld N. (2018). Tracking evolution of aromatase inhibitor resistance with circulating tumour DNA analysis in metastatic breast cancer. Ann. Oncol..

[21] Condorelli R., Spring L., O’shaughnessy J., Lacroix L., Bailleux C., Scott V., Dubois J., Nagy R.J., Lanman R.B., Iafrate A.J. (2018). Polyclonal RB1 mutations and acquired resistance to CDK 4/6 inhibitors in patients with metastatic breast cancer. Ann. Oncol..

[22] Oxnard G.R., Hu Y., Mileham K.F., Husain H., Costa D.B., Tracy P., Feeney N., Sholl L.M., Dahlberg S.E., Redig A.J. (2018). Assessment of resistance mechanisms and clinical implications in patients with EGFR T790M–positive lung cancer and acquired resistance to osimertinib. JAMA Oncol..

[23] Misale S., Yaeger R., Hobor S., Scala E., Janakiraman M., Liska D., Valtorta E., Schiavo R., Buscarino M., Siravegna G. (2012). Emergence of KRAS mutations and acquired resistance to anti-EGFR therapy in colorectal cancer. Nature.

[24] Schreuer M., Meersseman G., Van Den Herrewegen S., Jansen Y., Chevolet I., Bott A., Wilgenhof S., Seremet T., Jacobs B., Buyl R. (2016). Quantitative assessment of BRAF V600 mutant circulating cell-free tumor DNA as a tool for therapeutic monitoring in metastatic melanoma patients treated with BRAF/MEK inhibitors. J. Transl. Med..

[25] Tie J., Wang Y., Tomasetti C., Li L., Springer S., Kinde I., Silliman N., Tacey M., Wong H.-L., Christie M. (2016). Circulating tumor DNA analysis detects minimal residual disease and predicts recurrence in patients with stage II colon cancer. Sci. Transl. Med..

[26] Ignatiadis M., Litière S., Rothé F., Riethdorf S., Proudhon C., Fehm T., Silliman N., Tacey M., Wong H.L., Christie M. (2018). Trastuzumab versus observation for HER2 nonamplified early breast cancer with circulating tumor cells (EORTC 90091–10093, BIG 1–12, Treat CTC): A randomized phase II trial. Ann. Oncol..

[27] van Dalum G., Stam G.-J., Scholten L.F., Mastboom W.J., Vermes I., Tibbe A.G., De Groot M.R., Terstappen L.W. (2015). Importance of circulating tumor cells in newly diagnosed colorectal cancer. Int. J. Oncol..

[28] Krebs M.G., Sloane R., Priest L., Lancashire L., Hou J.-M., Greystoke A., Ward T.H., Ferraldeschi R., Andrew Hughes A., Clack G. (2011). Evaluation and prognostic significance of circulating tumor cells in patients with non–small-cell lung cancer. J. Clin. Oncol..

[29] Rossi G., Mu Z., Rademaker A.W., Austin L.K., Strickland K.S., Costa R.L.B., Nagy R.J., Zagonel V., Taxter T.J., Behdad A. (2018). Cell-free DNA and circulating tumor cells: Comprehensive liquid biopsy analysis in advanced breast cancer. Clin. Cancer Res..

[30] Cohen J.D., Li L., Wang Y., Thoburn C., Afsari B., Danilova L., Douville C., Ammar A., Javed A.A., Wong F. (2018). Detection and localization of surgically resectable cancers with a multi-analyte blood test. Science.

[31] Chan K.A., Woo J.K., King A., Zee B.C., Lam W.J., Chan S.L., Chan S.L., Chu S.W.I., Mak C., Tse I.O.L. (2017). Analysis of plasma Epstein–Barr virus DNA to screen for nasopharyngeal cancer. N. Engl. J. Med..

[32] Arneth B. (2018). Update on the types and usage of liquid biopsies in the clinical setting: A systematic review. BMC Cancer.

[33] Gandara D.R., Paul S.M., Kowanetz M., Schleifman E., Zou W., Li Y., Rittmeyer A., Fehrenbacher L., Otto G., Malboeuf C. (2018). Blood-based tumor mutational burden as a predictor of clinical benefit in non-small-cell lung cancer patients treated with atezolizumab. Nat. Med..

[34] Boutin A.T., Liao W.-T., Wang M., Hwang S.S., Karpinets T.V., Cheung H., Chu G.C., Jiang S., Hu J., Chang K. (2017). Oncogenic Kras drives invasion and maintains metastases in colorectal cancer. Genes Dev..

[35] Vidal J., Muinelo L., Dalmases A., Jones F., Edelstein D., Iglesias M., Edelstein D., Iglesias M., Orrillo M., Abalo A. (2017). Plasma ctDNA RAS mutation analysis for the diagnosis and treatment monitoring of metastatic colorectal cancer patients. Ann. Oncol..

[36] Montagut C., Argilés G., Ciardiello F., Poulsen T.T., Dienstmann R., Kragh M., Kopetz S., Lindsted T., Ding C., Vidal J. (2018). Efficacy of Sym004 in patients with metastatic colorectal cancer with acquired resistance to anti-EGFR therapy and molecularly selected by circulating tumor DNA analyses: A phase 2 randomized clinical trial. JAMA Oncol..

[37] Osumi H., Shinozaki E., Yamaguchi K., Zembutsu H. (2019). Clinical utility of circulating tumor DNA for colorectal cancer. Cancer Sci..

[38] Diehl F., Li M., Dressman D., He Y., Shen D., Szabo S., Diaz L.A., Goodman S.N., David K.A., Juhl H. (2005). Detection and quantification of mutations in the plasma of patients with colorectal tumors. Proc. Natl. Acad. Sci. USA.

[39] Alix-Panabières C., Pantel K. (2016). Clinical applications of circulating tumor cells and circulating tumor DNA as liquid biopsy. Cancer Discov..

[40] Cohen J.D., Javed A.A., Thoburn C., Wong F., Tie J., Gibbs P., Schmidt C.M., Yip-Schneider M.T., Allen P.J., Schattner M. (2017). Combined circulating tumor DNA and protein biomarker-based liquid biopsy for the earlier detection of pancreatic cancers. Proc. Natl. Acad. Sci. USA.

[41] Oshiro C., Kagara N., Naoi Y., Shimoda M., Shimomura A., Maruyama N., Shimazu K., Kim S.J., Noguchi S. (2015). PIK3CA mutations in serum DNA are predictive of recurrence in primary breast cancer patients. Breast Cancer Res. Treat..

[42] Otsuji K., Sasaki T., Tanaka A., Kunita A., Ikemura M., Matsusaka K., Tada k., Masashi Fukayama M., Seto Y. (2017). Use of droplet digital PCR for quantitative and automatic analysis of the HER2 status in breast cancer patients. Breast Cancer Res. Treat..

[43] Tantiwetrueangdet A., Panvichian R., Wongwaisayawan S., Sueangoen N., Lertsithichai P. (2018). Droplet digital PCR using HER2/EIF2C1 ratio for detection of HER2 amplification in breast cancer tissues. Med. Oncol..

[44] Nicolini A., Ferrari P., Duffy M.J. (2018). Prognostic and predictive biomarkers in breast cancer: Past, present and future. Semin. Cancer Biol..

[45] Board R.E., Wardley A.M., Dixon J.M., Armstrong A.C., Howell S., Renshaw L., Donald E., Greystoke A., Ranson M., Hughes A. (2010). Detection of PIK3CA mutations in circulating free DNA in patients with breast cancer. Breast Cancer Res. Treat..

[46] Takeshita T., Yamamoto Y., Yamamoto-Ibusuki M., Inao T., Sueta A., Fujiwara S., Omoto Y., Hirotaka Iwase H. (2015). Prognostic role of PIK 3 CA mutations of cell-free DNA in early-stage triple negative breast cancer. Cancer Sci..

[47] Baselga J., Im S.-A., Iwata H., Clemons M., Ito Y., Awada A., Chia S., Jagiello-Gruszfeld A., Pistilli B., Tseng L.M. (2016). Abstract S6–01: PIK3CA status in circulating tumor DNA (ctDNA) predicts efficacy of buparlisib (BUP) plus fulvestrant (FULV) in postmenopausal women with endocrine-resistant HR+/HER2–advanced breast cancer (BC): First results from the randomized, phase III BELLE-2 trial. Cancer Res..

[48] Jelovac D., Beaver J.A., Balukrishna S., Wong H.Y., Toro P.V., Cimino-Mathews A., Cimino-Mathews A., Argani P., Stearns V., Jacobs L. (2014). A PIK3CA mutation detected in plasma from a patient with synchronous primary breast and lung cancers. Hum. Pathol..

[49] Perkins G., Lu H., Garlan F., Taly V. (2018). Droplet-based digital PCR: Application in cancer research. Exp. Rev. Mol. Diagn..

[50] Litwin M.S., Tan H.-J. (2017). The diagnosis and treatment of prostate cancer: A review. JAMA.

[51] Danila D.C., Samoila A., Patel C., Schreiber N., Herkal A., Anand A., Bastos D., Heller G., Fleisher M., Scher H.I. (2016). Clinical validity of detecting circulating tumor cells by AdnaTest assay compared to direct detection of tumor mRNA in stabilized whole blood, as a biomarker predicting overall survival for metastatic castration-resistant prostate cancer patients. Cancer J..

[52] De Bono J.S., Scher H.I., Montgomery R.B., Parker C., Miller M.C., Tissing H., Doyle G.V., Terstappen L.W., Pienta K.J., Raghavan D. (2008). Circulating tumor cells predict survival benefit from treatment in metastatic castration-resistant prostate cancer. Clin. Cancer Res..

[53] Goldkorn A., Ely B., Tangen C.M., Tai Y.C., Xu T., Li H., Twardowski P., Veldhuizen P.J., Agarwal N., Carducci M.A. (2015). Circulating tumor cell telomerase activity as a prognostic marker for overall survival in SWOG 0421: A phase III metastatic castration resistant prostate cancer trial. Int. J. Cancer..

[54] Scher H.I., Jia X., de Bono J.S., Fleisher M., Pienta K.J., Raghavan D., Heller G. (2009). Circulating tumour cells as prognostic markers in progressive, castration-resistant prostate cancer: A reanalysis of IMMC38 trial data. Lancet Oncol..

[55] Logothetis C.J., Basch E., Molina A., Fizazi K., North S.A., Chi K.N., Jones R.J., Goodman O.B., Mainwarning P.N., Sternberg C.N. (2012). Effect of abiraterone acetate and prednisone compared with placebo and prednisone on pain control and skeletal-related events in patients with metastatic castration-resistant prostate cancer: Exploratory analysis of data from the COU-AA-301 randomised trial. Lancet Oncol..

[56] Wyatt A.W., Azad A.A., Volik S.V., Annala M., Beja K., McConeghy B., Haegert A., Warner E.W., Mo F., Brahmbhatt S. (2016). Genomic alterations in cell-free DNA and enzalutamide resistance in castration-resistant prostate cancer. JAMA Oncol..

[57] Kong D., Sethi S., Li Y., Chen W., Sakr W.A., Heath E. (2015). Androgen receptor splice variants contribute to prostate cancer aggressiveness through induction of EMT and expression of stem cell marker genes. Prostate.

[58] Uo T., Plymate S.R., Sprenger C.C. (2018). The potential of AR-V7 as a therapeutic target. Exp. Opin. Ther. Targ..

[59] Arnold M., Holterhues C., Hollestein L., Coebergh J., Nijsten T., Pukkala E., Holleczek B., Tryggvadóttir L., Comber H., Bento M.J. (2014). Trends in incidence and predictions of cutaneous melanoma across Europe up to 2015. J. Eur. Acad. Dermatol. Venereol..

[60] Lee R.J., Gremel G., Marshall A., Myers K., Fisher N., Dunn J., Dhomen N., Corrie P.G., Middleton M.R., Lorigan P. (2017). Circulating tumor DNA predicts survival in patients with resected high-risk stage II/III melanoma. Ann. Oncol..

[61] Gonzalez-Cao M., Mayo-de-las-Casas C., Molina-Vila M.A., De Mattos-Arruda L., Muñoz-Couselo E., Manzano J.L., Cortes J., Berros J.P., Drozdowskyj A., Sanmamed M. (2015). BRAF mutation analysis in circulating free tumor DNA of melanoma patients treated with BRAF inhibitors. Melanoma Res..

[62] McEvoy A.C., Warburton L., Al-Ogaili Z., Celliers L., Calapre L., Pereira M.R., Khattak M.A., Meniawy T.M., Millward M., Ziman M. (2018). Correlation between circulating tumour DNA and metabolic tumour burden in metastatic melanoma patients. BMC Cancer.

[63] Valpione S., Gremel G., Mundra P., Middlehurst P., Galvani E., Girotti M.R., Lee R.J., Garner G., Dhomen N., Lorigan P.C. (2018). Plasma total cell-free DNA (cfDNA) is a surrogate biomarker for tumour burden and a prognostic biomarker for survival in metastatic melanoma patients. Eur. J. Cancer.

[64] Bidard F.C., Madic J., Mariani P., Piperno-Neumann S., Rampanou A., Servois V., Cassoux N., Desjardins L., Milder M., Vaucher I. (2014). Detection rate and prognostic value of circulating tumor cells and circulating tumor DNA in metastatic uveal melanoma. Int. J. Cancer.

